# Genetic Markers Can Predict Chondrogenic Differentiation Potential in Bone Marrow-Derived Mesenchymal Stromal Cells

**DOI:** 10.1155/2018/9530932

**Published:** 2018-10-10

**Authors:** Masami Kanawa, Akira Igarashi, Katsumi Fujimoto, Yukihito Higashi, Hidemi Kurihara, Masaru Sugiyama, Tania Saskianti, Yukio Kato, Takeshi Kawamoto

**Affiliations:** ^1^Natural Science Center for Basic Research and Development, Hiroshima University, Hiroshima 734-8533, Japan; ^2^Department of Advanced Technology and Development, BML, Inc., Saitama 350-1101, Japan; ^3^Department of Dental and Medical Biochemistry, Graduate School of Biomedical & Health Sciences, Hiroshima University, Hiroshima 734-8533, Japan; ^4^Department of Molecular Biology and Biochemistry, Graduate School of Biomedical & Health Sciences, Hiroshima University, Hiroshima 734-8533, Japan; ^5^Research Center for Radiation Genome Medicine, Research Institute for Radiation Biology and Medicine, Hiroshima University, Hiroshima 734-8533, Japan; ^6^Departments of Periodontal Medicine, Graduate School of Biomedical & Health Sciences, Hiroshima University, Hiroshima 734-8533, Japan; ^7^Departments of Public Oral Health, Graduate School of Biomedical & Health Sciences, Hiroshima University, Hiroshima 734-8533, Japan; ^8^Department of Pediatric Dentistry, Faculty of Dental Medicine, Universitas Airlangga, Surabaya 60132, Indonesia; ^9^Writing Center, Hiroshima University, Higashi-Hiroshima 739-8512, Japan

## Abstract

The precise predictions of the differentiation direction and potential of mesenchymal stromal cells (MSCs) are an important key to the success of regenerative medicine. The expression levels of fate-determining genes may provide tools for predicting differentiation potential. The expression levels of 95 candidate marker genes and glycosaminoglycan (GAG) contents after chondrogenic induction in 10 undifferentiated ilium and 5 jaw MSC cultures were determined, and their correlations were analyzed. The expression levels of eight genes before the induction of chondrogenic MSC differentiation were significantly correlated with the GAG levels after induction. Based on correlation patterns, the eight genes were classified into two groups: group 1 genes (*AURKB*, *E2F1*, *CDKN2D*, *LIF*, and *ACLY*), related to cell cycle regulation, and group 2 genes (*CD74*, *EFEMP1*, and *TGM2*), involved in chondrogenesis. The expression levels of the group 2 genes were significantly correlated with the ages of the cell donors. The expression levels of *CDKN2D*, *CD74*, and *TGM2* were >10-fold higher in highly potent MSCs (ilium MSCs) than in MSCs with limited potential (jaw MSCs). Three-dimensional (3D) scatter plot analyses of the expression levels of these genes showed reduced variability between donors and confirmed predictive potential. These data suggest that group 2 genes are involved in age-dependent decreases in the chondrogenic differentiation potential of MSCs, and combined 3D analyses of the expression profiles of three genes, including two group 2 genes, were predictive of MSC differentiation potential.

## 1. Introduction

Mesenchymal stromal cells (MSCs) can differentiate into various cell types, including osteoblasts, chondrocytes, or adipocytes; therefore, they are promising as regenerative medicine [1–4]. MSCs are usually obtained using bone marrow aspirated from the iliac crest. Recently, we developed another method to obtain MSCs from jaw bone marrow collected during wisdom tooth extraction [5], a surgery that several young adults undergo. The differentiation potential of MSCs varies depending on the tissue sources and the physical conditions of donors [6–9]. Accordingly, we previously demonstrated that MSCs from jaw bone marrow have poor chondrogenic differentiation capacity, although they have high osteogenic differentiation capacity, as seen in ilium MSCs [5]. In another study, we showed that chondrogenic differentiation potential of MSCs from ilium bone marrow depends on the age of cell donors [10].

Predicting the differentiation direction of MSCs is a crucial determinant of clinical outcomes of regenerative medicine, and several cell surface markers have been identified as predictors of such capabilities. CD105^+^, CD146^+^, CD271^+^, or ROR2^+^ MSCs have enhanced capacity for chondrogenic differentiation [11–14]. CD146^+^ MSCs also have greater therapeutic potential than CD146^−^ cells [15]. However, the utility of these cell surface markers has not yet been established. Hence, in addition to cell surface markers, gene expression patterns may provide a strategy for predicting the differentiation potential of MSCs.

Recently, we produced a TaqMan low-density array comprising real-time PCR probes and the primers for 95 marker candidate genes that were selected from microarray analyses of 17,703 genes [16]. Because these 95 genes showed higher expression levels in MSCs than in fibroblasts, we predicted that some of these genes may serve as MSC markers for identifying cells having high potential for differentiation into specific cell types such as chondrocytes.

In the present study, we aimed to find prediction markers to select potent MSCs by comparing gene expression profiles and differentiation levels. Accordingly, we determined the expression levels of 95 marker candidate genes in undifferentiated MSCs from various donors and analyzed the correlation between the expression and glycosaminoglycan (GAG) protein levels in MSCs after induction of chondrogenic differentiation. The mRNA levels of eight genes were strongly correlated with MSC potency, as indicated by GAG production.

## 2. Materials and Methods

### 2.1. Cells

Human bone marrow MSCs were isolated from patients at the Hiroshima University Hospital and were cultured with the approval of the Hiroshima University Ethics Committee, as described previously [10, 16]. Ilium MSCs were isolated from 10 patients aged 25, 39, 53, 55, 59, 61, 63, 64, 65, and 81 years, and jaw MSCs were collected from 5 patients aged 20, 28, 36, 36, and 63 years [10, 16]. The donor ID numbers and ages are listed in Table S1 in Supplementary Materials.

### 2.2. Chondrogenic Differentiation of MSCs

MSCs from fourth-passage cultures were seeded at 2.5 × 10^5^ cells in 15 mL centrifuge tubes for pellet culture in a chondrogenic differentiation medium and were maintained for 28 days as described previously [10, 17]. The GAG contents were then measured using a sulfated GAG assay kit (Biocolor), according to the manufacturer's instructions. Data were normalized by the amounts of genomic DNA determined using PicoGreen fluorescence assays (Invitrogen).

### 2.3. Quantitative RT-PCR

Total RNA was isolated from confluent third-passage cultures using the RNeasy Mini Kit (Qiagen) as described previously [16]. First-strand cDNA was synthesized using ReverTra Ace-*α* (Toyobo), and real-time quantitative PCR was performed using the ABI Prism 7900 Sequence Detection System (Applied Biosystems) with a TaqMan low-density array (Applied Biosystems), which contains TaqMan probe and primer sets (TaqMan Gene Expression Assays) for 95 genes. The 95 genes were selected because their expression levels in ilium or jaw MSCs were more than two-fold higher than those in fibroblasts on microarray analyses [16]. The probe set IDs of TaqMan probe and primer sets for *ACLY*, *AURKB*, *CD74*, *CDKN2D*, *E2F1*, *EFEMP1*, *LIF*, and *TGM2* are provided in Table 1. The genes examined in this study are listed in Table S1 in Supplementary Materials with their mRNA expression levels relative to that of *β*-actin.

### 2.4. Statistical Analysis

Statistical analyses were performed using SPSS Statistics for Windows, version 24.0. (IBM Corp.). Comparisons were made using Student's *t*-tests when comparing two experimental groups; correlations between mRNA expression levels and GAG contents, donor ages, or the mRNA expression levels of other genes were identified using Pearson correlation coefficients. The coefficient between the mRNA levels of group 2 genes and donor ages were calculated by linear regression analysis. Differences and correlations were considered significant when *P* < 0.05.

### 2.5. Cluster Analysis

Correlations between the mRNA expression levels of *ACLY*, *AURKB*, *CD74*, *CDKN2D*, *E2F1*, *EFEMP1*, *LIF*, and *TGM2* were evaluated using hierarchical cluster analyses with the nearest-neighbor algorithm and are presented in a dendrogram that was generated using SPSS version 24.0.

### 2.6. Three-Dimensional (3D) Scatter Plot Analysis

3D scatter plots were constructed using SPSS version 24.0. In this analysis, the relative mRNA expression levels of *CDKN2D*, *CD74*, and *TGM2* in MSCs from 15 donors were recalculated relative to maximum values of 100 for each gene, and the distances between each point and the origin were then calculated.

## 3. Results

### 3.1. Chondrogenic Differentiation Potential of Ilium and Jaw MSCs

**Figure 1:**
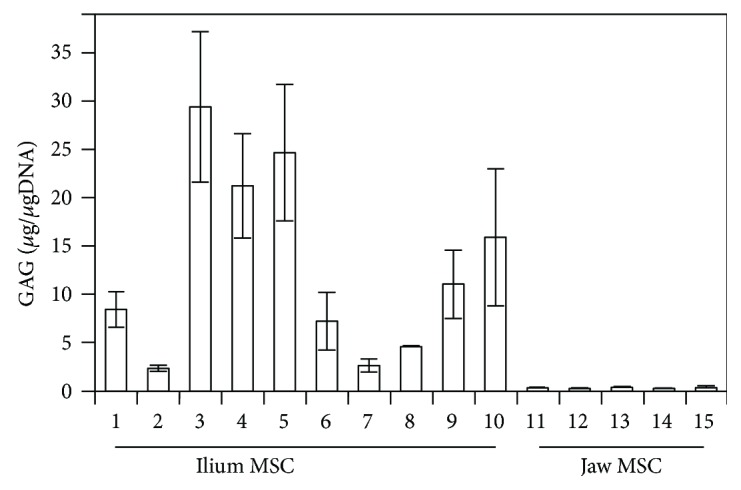
Chondrogenic differentiation potential of MSCs from ilium and jaw bone marrow. Chondrogenic differentiation levels were evaluated according to GAG contents at 28 days after induction. Data are presented as mean GAG contents normalized by genomic DNA amounts ± standard errors of the mean from triplicate cultures; 1–10: ilium MSCs; 11–15: jaw MSCs.

The potential and direction of differentiation vary depending on the source of the MSCs. In our previous study, we found that jaw MSCs have modest chondrogenic differentiation capacity compared with MSCs from the ilium [5, 16]. Accordingly, the GAG contents of the pellet cultures of ilium MSCs (lanes 1–10) were much higher (2.3 to 29.4 *μ*g) than those of the pellet cultures of jaw MSCs (lanes 11–15; <0.3 *μ*g) at 28 days after induction of chondrogenesis (Figure 1). However, the GAG contents varied widely even among ilium MSC cultures.

### 3.2. Correlations between Gene Expression Levels in Undifferentiated MSCs and GAG Contents after Induction of Chondrogenesis

The differences in chondrogenic differentiation potential between the ilium and jaw MSCs may help identify marker genes that can predict the MSC potential before induction. Thus, we assessed the correlations between gene expression levels in 15 MSC cultures (10 from ilium tissues and 5 from jaw tissues) and their differentiation levels after chondrogenic induction. We quantified the mRNA levels of 95 marker candidate genes using TaqMan low-density arrays [16] and analyzed the correlation between these levels and GAG contents. The mRNA expression patterns of eight of the 95 genes were significantly correlated with the GAG contents after induction (Table S1 in Supplementary Materials and Table 1); therefore, we selected these as candidate predictors of differentiation potential.

### 3.3. Correlation Analysis of Expression Levels of Eight Genes with Predictive Potential

**Figure 2:**
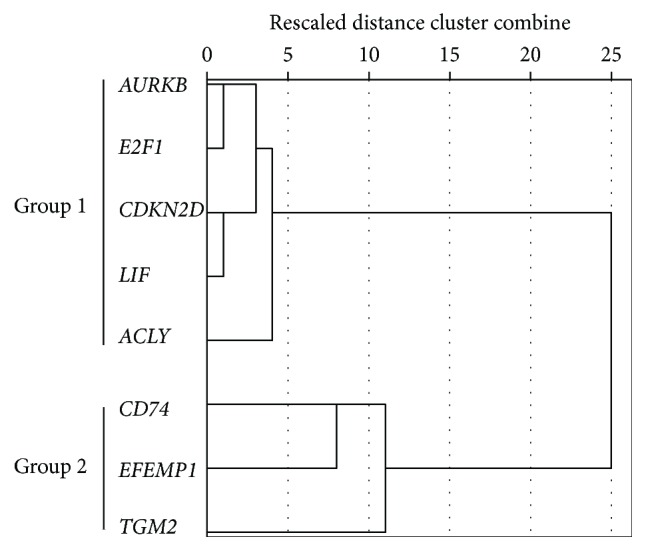
Dendrogram of the expression profiles of eight candidate prediction marker genes. Hierarchical cluster analysis of gene expression levels was performed using the nearest-neighbor algorithm of SPSS. The horizontal dendrogram shows rescaled cumulative distances of clusters. The eight identified genes were classified into groups 1 and 2 according to the gene expression profiles.

To further examine the relationships between the identified eight genes, we correlated expression profiles in 15 MSC cultures (Table 2). These analyses showed significant correlations in several combinations, presumably reflecting common regulatory mechanisms. To confirm these assumptions, we performed hierarchical cluster analyses using the nearest-neighbor algorithm and generated a dendrogram (Figure 2) that represents the overall relationships between the eight genes. Based on these correlation patterns, *AURKB*, *E2F1*, *CDKN2D*, *LIF*, and *ACLY* were allocated to group 1 and *CD74*, *EFEMP1*, and *TGM2* were allocated to group 2, suggesting the presence of two predominant signaling pathways that determine chondrogenic differentiation potential.

### 3.4. Comparisons of Gene Expression Levels between Ilium and Jaw MSCs

**Figure 3:**
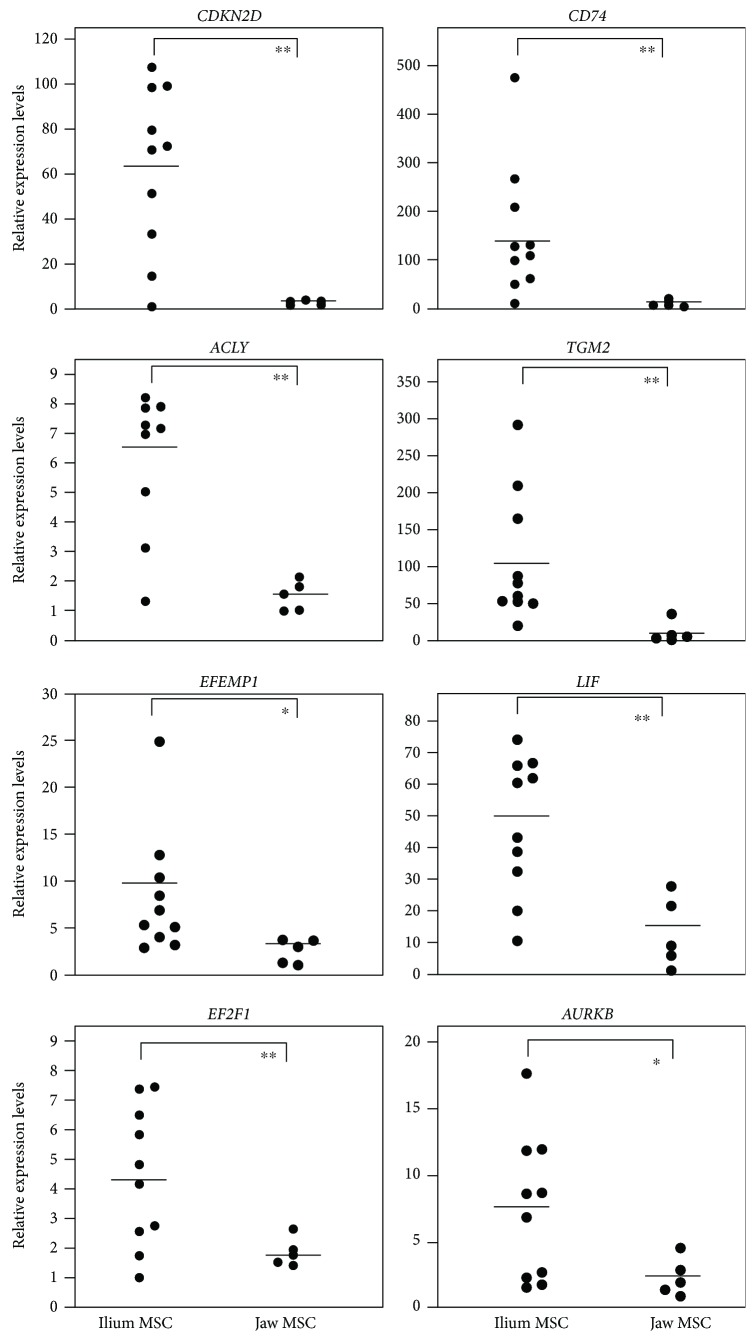
Comparisons of the expression levels of the eight candidate predictors of chondrogenesis between ilium MSCs (*n* = 10) and jaw MSCs (*n* = 5). Bars indicate mean values for each group; ^∗^ *P* < 0.05, ^∗∗^ *P* < 0.01; Student's *t*-test.

Because the GAG contents were consistently lower in jaw MSCs than in those from the ilium, we compared the expression levels of the eight identified genes in MSCs from both tissues. These analyses revealed significantly higher mRNA expression levels of all eight genes in ilium MSCs than in jaw MSCs (Figure 3). Moreover, the expression levels of *CDKN2D*, *CD74*, and *TGM2* were 22-, 18-, and 10-fold higher, respectively, in ilium MSCs than in jaw MSCs. Thus, we selected these three genes as potential markers for further combined analyses.

### 3.5. Combined 3D Analysis of *CDKN2D*, *CD74*, and *TGM2* Expression Levels

**Figure 4:**
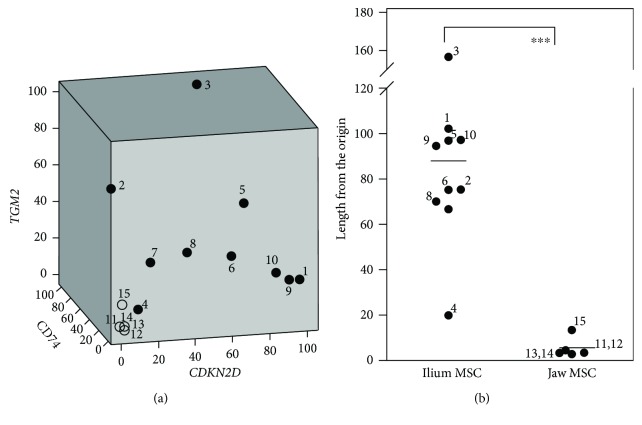
Combined 3D analysis of mRNA expression levels. (a) The scatter plot of *CDKN2D* (*x*-axis), *CD74* (*y*-axis), and *TGM2* (*z*-axis) mRNA expression levels was constructed using SPSS. The relative mRNA expression levels in ilium (nos. 1–10) and jaw MSCs (nos. 11–15) are presented relative to maximum values of 100. (b) Mean distances between each point and the origin (bars) are shown for ilium and jaw MSCs. The donor ID numbers in the graphs correspond to those shown in Table S1 in Supplementary Materials; ^∗∗∗^ *P* < 0.001; Student's *t*-test.

**Figure 5:**
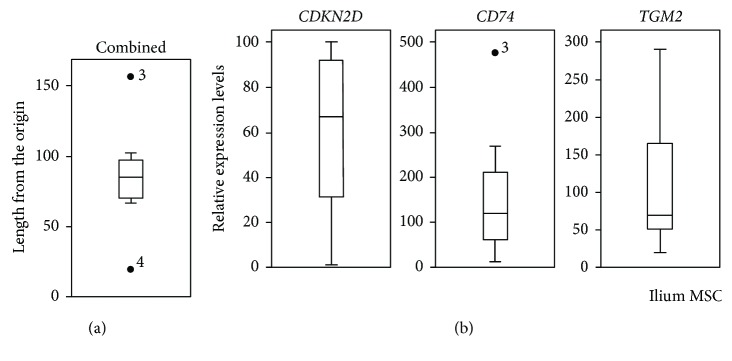
Comparisons of gene expression levels in ilium MSCs (*n* = 10). (a) Box plots show distances from the origin in combined 3D analysis of *CDKN2D*, *CD74*, and *TGM2*, as shown in Figure 4(a). (b) Box plots of *CDKN2D*, *CD74*, and *TGM2* expression levels are presented with medians (bisecting line), interquartile ranges (box), 1.5-times interquartile ranges (whiskers), and outliers (dots). Outliers are numbered according to the donor IDs.

Although the *CDKN2D*, *CD74*, and *TGM2* expression levels were much higher in ilium MSCs than in jaw MSCs, these considerably varied among the cultures of ilium MSCs. Thus, to predict MSC potential, we performed combined 3D analyses of expression levels and generated a 3D scatter plot for the three genes (Figure 4(a)). Distances from the origin for 9 out of 10 ilium MSCs (66.6 to 156.6) were greater than those for all jaw MSCs (2.8 to 13.4), whereas that for ilium MSC-4 was only 19.9 (Figure 4(b)). In addition, variations in combined expression data (Figure 5(a)) for all three genes were much smaller than for individual genes (Figure 5(b)). Hence, these combined 3D analyses may provide a more useful standard for predicting chondrogenic differentiation.

### 3.6. Negative Correlations between Gene Expression Levels and Donor Ages

**Figure 6:**
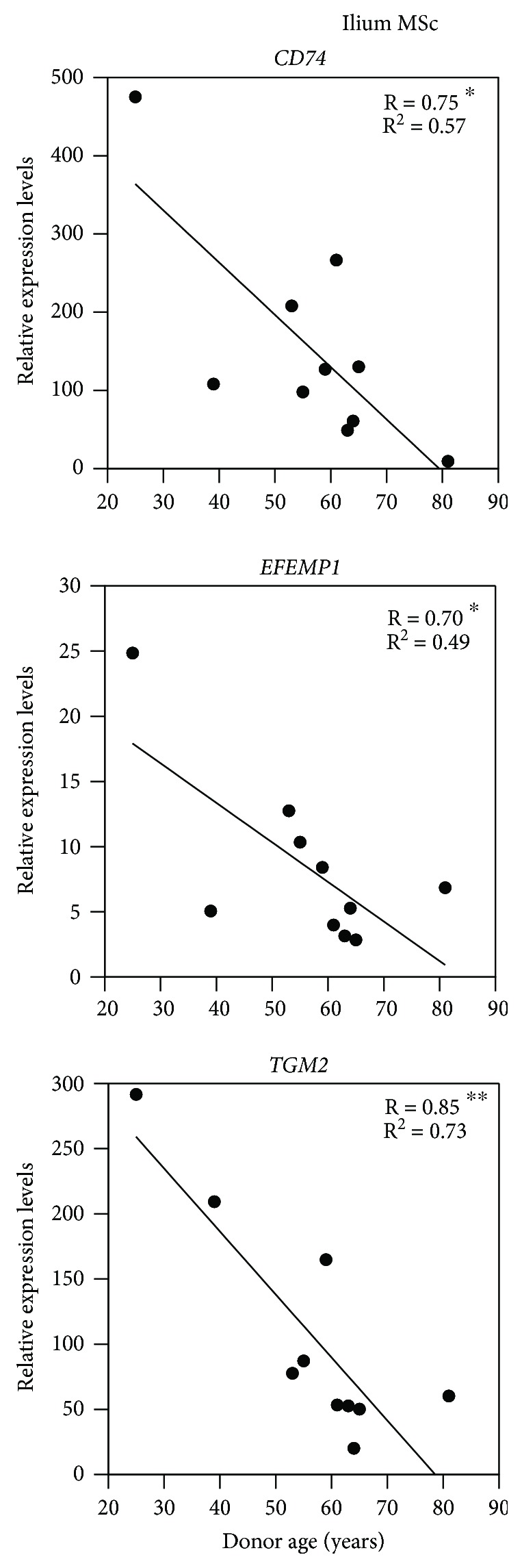
Regression analyses of the expression levels of the group 2 genes *CD74*, *EFEMP1*, and *TGM2* in ilium MSCs and the donor ages. *n* = 10; ^∗^ *P* < 0.05, ^∗∗^ *P* < 0.01. R: multiple correlation coefficient; R^2^: coefficient of determination.

We previously identified age-dependent decreases in chondrogenic differentiation potential of MSCs [10]. Thus, to evaluate the effects of age on the expression levels of the present eight genes with predictive potential, we correlated donor ages and mRNA expression levels in MSCs. The expression levels of the group 2 genes *CD74*, *EFEMP1*, and *TGM2* were negatively correlated with donor ages (Table 3 and Figure 6), whereas no significant correlations were found for group 1 genes, suggesting that group 2 genes play predominant roles in age-dependent MSC chondrogenic differentiation potential.

## 4. Discussion

The predictions of MSC differentiation direction and potential are critical for clinical applications because the ensuing multipotency varies among donors. Although the factors that dictate differentiation potential into specific cell types remain poorly understood, we found that after chondrogenic induction, GAG contents were consistently low in pellet cultures of all MSCs from jaw tissues compared with those from ilium MSCs. Further, we attempted to identify the genes expressed at high levels in MSCs fated to produce large amounts of GAG but not in those fated to produce only small amounts of GAG after chondrogenic induction.

To identify markers of chondrocyte differentiation potential in MSCs, we correlated the gene expression in undifferentiated MSC monolayer cultures with GAG production in MSC pellet cultures after differentiation into chondrocytes. In these expression analyses, eight genes were significantly correlated with GAG contents, and these were classified into two groups according to their transcription profiles. These cluster analyses suggest common transcriptional regulatory mechanisms. Accordingly, the group 1 genes *AURKB* [18], *E2F1* [19–21], *CDKN2D* [22], *LIF* [23, 24], and *ACLY* [25] participate in cell cycle regulation, whereas the group 2 genes *EFEMP1* [26, 27], *CD74* [24, 28–30], and *TGM2* [31, 32] are involved in the regulation of chondrogenesis.

EFEMP1 (also known as fibulin-3), an ECM protein, is specifically expressed in cartilage and acts as a negative regulator of chondrogenesis [26]. Accordingly, overexpression of *EFEMP1* reportedly suppressed the expression of the chondrogenic proteins SOX9, type II collagen, and aggrecan, whereas knockdown of *EFEMP1* facilitated chondrogenesis [27]. CD74 is a receptor for macrophage migration inhibitory factor [33], which upregulates SOX9 expression [34]. TGM2 catalyzes the crosslinking of proteins such as fibronectin [31, 32] and thereby plays important roles in chondrogenesis [35].

Among the eight genes identified herein, the mean *CDKN2D*, *CD74*, and *TGM2* mRNA expression levels were more than 10-fold higher in ilium MSCs than in jaw MSCs. Although these varied widely between ilium MSC cultures, combined 3D analyses of these three genes showed promising results with very low variations and may represent a novel strategy for determining MSC differentiation potential.

MSCs are multipotent, and their fates are likely unpredictable, even if their potential for differentiation can be estimated. The present 3D combined analyses may offer promising assessments of differentiation potential, although the combined expression level in the MSC-4 culture was relatively low, despite the high GAG contents. To compensate for this discrepancy, we performed additional two-dimensional analysis of *CDKN2D* and *CD74* (Figure S1 in Supplementary Materials), which showed much greater combined expression levels in ilium MSC-4 (no. 4) cultures than in jaw MSCs (nos. 11–15), similar combined expression to that in ilium MSC-2 (no. 2) cultures, but much lower combined expression than in the other ilium MSC cultures (nos. 1, 3, and 5–10). Although this approach is sensitive to the expression of multiple genes, further studies of multiple MSC cultures from various tissues at various passages are required and could include fat- and synovium-derived MSCs.

In the present study, we used only 10 ilium and 5 jaw MSC lines owing to limited availability of MSC lines. Variations of the expression levels of the eight genes in ilium MSCs were much larger than those in jaw MSCs. Fortunately, we could examine 10 ilium MSC lines compared with 5 jaw MSC lines. In future research, the number of MSC lines should be increased to confirm our present results.

Although donor age strongly influences the potential for chondrogenic differentiation of MSCs, we previously showed that it is not a predictor of osteogenic or adipogenic differentiation [10]. In this study, the expression levels of the group 2 genes *EFEMP1*, *CD74*, and *TGM2* were negatively correlated with the donor ages, potentially indicating their characterized roles in chondrogenesis. Collectively, these findings suggest that the group 2 genes are responsible for age-dependent chondrogenic differentiation. In agreement, *EFEMP1* expression in articular cartilage was previously shown to decrease in an age-dependent manner [27].

Recently, Bertolo et al. [36] demonstrated marked decreases in the chondrogenic differentiation potential of MSCs after passage 7. In their study concerning 84 senescence-related genes, the expression levels of 14 genes, including *E2F1* and *CDKN2D*, were more than two-fold lower at passage 9 than at passage 3. As shown herein, these data suggest that high-level expression of cell cycle regulators such as *E2F1* and *CDKN2D* is a requirement for high chondrogenic differentiation potential. In a previous study, we also evaluated the expression levels of 20 MSC marker genes, including *LIF*, *CD74*, and *TGM2*, from passages 1 to 10 [16]. Notably, *LIF* maintained similar expression levels from passages 1 to 10, but the expression level of *CD74* was upregulated at passage 10. Further, the expression level of *TGM2* at passage 1 is much higher than those from passages 2 to 10. Thus, the expression levels of the marker genes found in this study showed various patterns. The relationship between expression levels of the eight genes and decreases in the chondrogenic differentiation potential after several passages should be analyzed in future research.

We previously identified several genes with potential as MSC markers [16], and among these, *TGM2* and *LIF* expressions were downregulated after the induction of chondrogenic differentiation [37]. Thus, markers of differentiation potential likely differ from markers of differentiation such as type II collagen and aggrecan, which are upregulated only after chondrogenic differentiation [38]. Therefore, while the markers identified herein indicate MSC potential, their expression levels are unlikely to distinguish between the degrees of differentiation.

Several cell surface markers, including CD105, CD146, CD271, and receptor tyrosine kinase-like orphan receptor 2 (ROR2), have been considered as markers of MSCs with high potential for chondrocyte differentiation [11–14]. However, to evaluate the expression levels of cell surface markers, we need experiments using fluorescence-activated cell sorter (FACS) analysis, which uses suitable antigens for detecting cell surface molecules and single cells detached from culture dishes. The nature of MSCs detached from the dishes may differ from that of cells attached to the dishes. In addition, evaluation using FACS analysis is not quantitative, although FACS can be used to select a subpopulation that expresses high levels of a cell surface marker. Among these cell surface markers, the CD146 (MCAM) gene was included in the 95 genes evaluated in this study (Table S1 in Supplementary Materials). CD146 showed a similar expression pattern as the eight genes identified in this study. Its expression level in ilium MSCs was significantly higher than that in jaw MSCs (data not shown), although the correlation of CD146 expression with GAG production was not significant, suggesting that the eight genes are more reliable markers than cell surface markers such as CD146. Thus, measuring the prediction marker expression levels in cells attached to the dishes could be desirable for estimating the differentiation potential of MSCs.

## 5. Conclusions

The present 3D expression analyses of predictive marker genes offer a novel strategy for assessing MSC differentiation potential and could form the basis for predicting clinical outcomes of MSC therapy.

## Figures and Tables

**Table 1:** Candidate prediction marker genes showing significant positive correlation between their expression levels in undifferentiated MSCs and GAG contents after chondrogenic induction.

Gene	Full name	Probe set ID	*r*
*ACLY*	ATP citrate lyase	Hs00153764_m1	0.531^∗^
*AURKB*	Aurora kinase B	Hs00177782_m1	0.570^∗^
*CD74*	CD74 molecule	Hs00269961_m1	0.565^∗^
*CDKN2D*	Cyclin-dependent kinase inhibitor 2D	Hs00176481_m1	0.577^∗^
*E2F1*	E2F transcription factor 1	Hs00153451_m1	0.566^∗^
*EFEMP1*	EGF containing fibulin extracellular matrix protein 1	Hs00251661_m1	0.637^∗^
*LIF*	Leukemia inhibitory factor	Hs00171455_m1	0.603^∗^
*TGM2*	Transglutaminase 2	Hs00190278_m1	0.603^∗^

*r*: Pearson correlation coefficient; ^∗^ *P* < 0.05.

**Table 2:** Correlation coefficients for expression levels of eight candidate predictive marker genes.

Gene	*AURKB*	*E2F1*	*CDKN2D*	*LIF*	*ACLY*	*CD74*	*EFEMP1*	*TGM2*
*AURKB*	**1.000**							
*E2F1*	**0.940** ^∗∗^	**1.000**						
*CDKN2D*	**0.825** ^∗∗^	**0.899** ^∗∗^	**1.000**					
*LIF*	**0.836** ^∗∗^	**0.905** ^∗∗^	**0.933** ^∗∗^	**1.000**				
*ACLY*	**0.711** ^∗∗^	**0.811** ^∗∗^	**0.893** ^∗∗^	**0.889** ^∗∗^	**1.000**			
*CD74*	0.244	0.406	0.314	0.401	0.571^∗^	**1.000**		
*EFEMP1*	0.396	0.483	0.366	0.369	0.502	**0.827** ^∗∗^	**1.000**	
*TGM2*	0.343	0.431	0.315	0.419	0.494	**0.751** ^∗∗^	**0.785** ^∗∗^	**1.000**

*r*: Pearson correlation coefficient; ^∗^ *P* < 0.05, ^∗∗^ *P* < 0.01. Strong Pearson correlation coefficient: >0.75.

**Table 3:** Correlations between expression levels of candidate predictive marker genes and donor ages.

Group	Gene	*r*
1	*AURKB*	0.268
1	*E2F1*	0.083
1	*CDKN2D*	0.363
1	*LIF*	0.218
1	*ACLY*	0.116
2	*CD74*	−0.754^∗^
2	*EFEMP1*	−0.702^∗^
2	*TGM2*	−0.852^∗∗^

*r*: Pearson correlation coefficients; ^∗^ *P* < 0.05, ^∗∗^ *P* < 0.01.

## Data Availability

The data used to support the findings of this study are available from the corresponding author upon request.

## Supplementary Materials

Supplementary Materials. Figure S1: combined two-dimensional analysis of CD74 and CDKN2D mRNA expression levels. Table S1: correlation coefficients for 95 gene expression levels in undifferentiated MSC and GAG contents after chondrogenic induction, along with donor IDs and ages.
