# Supplementary material for: Genetic Markers Can Predict Chondrogenic Differentiation Potential in Bone Marrow-Derived Mesenchymal Stromal Cells
Source: Stem Cells Int. 2018 Oct 10;2018:9530932. doi: 10.1155/2018/9530932 (PMC6199884; doi:10.1155/2018/9530932)
Supplement: Supplementary Materials. — Figure S1: combined two-dimensional analysis of CD74 and CDKN2D mRNA expression levels. Table S1: correlation coefficients for 95 gene expression levels in undifferentiated MSC and GAG contents after chondrogenic induction, along with donor IDs and ages. [file 9530932.f1.pdf]

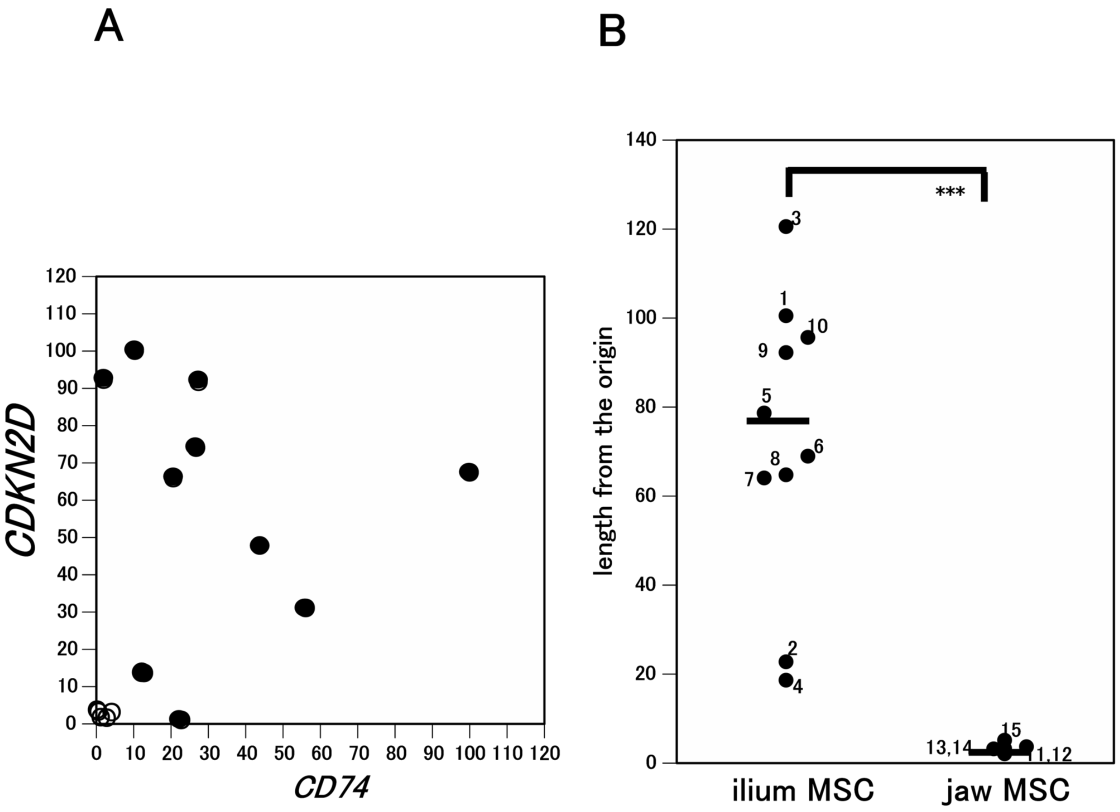

Figure S1: Combined two-dimensional analysis of CD74 and CDKN2D mRNA expression levels are presented in a scatter plot (A). The relative mRNA expression levels in ilium MSCs (closed circles) and jaw MSCs (open circles) are presented relative to maximum values of 100. (B) Distances between data points and the origin are shown in panel A; ilium MSCs (Nos. 1–10) and jaw MSCs (Nos. 11–15). The mean values for each group are indicated as bars. The numbers in the graphs correspond to the donor IDs in Supplementary Table I; ***$P < 0.001$; Student's *t*-test.

Table S1: Correlation coefficients for gene expression levels in undifferentiated MSC and GAG contents after chondrogenic induction

donor ID number	1	2	3	4	5	6	7	8	9	10	11	12	13	14	15	r
**donor age (years)**	63	39	25	64	59	55	61	53	81	65	63	36	36	20	28	
**GAG (μg/μgDNA)**	8.41	2.34	29.40	21.27	24.66	7.22	2.64	4.56	11.07	15.91	0.17	0.13	0.29	0.18	0.26	
ACLY	7.90	3.13	7.91	1.33	7.86	5.03	6.97	7.28	8.21	7.17	1.00	1.82	2.15	1.03	1.57	0.531 *
ACVR2B	7.77	3.86	5.30	1.00	5.47	3.53	3.35	2.96	5.07	4.86	16.99	28.18	18.83	11.96	12.18	-0.520 *
ADD3	2.96	2.84	2.58	1.00	2.45	1.87	3.09	2.77	2.35	3.84	7.75	10.99	5.50	12.78	8.01	-0.588 *
AMD1	5.35	2.05	3.95	1.00	3.93	3.25	4.66	4.34	6.22	6.80	2.73	3.23	4.31	2.50	4.28	0.087
AMFR	4.90	2.06	3.71	1.00	3.69	1.76	2.66	4.88	3.29	4.04	5.52	8.09	6.70	7.28	4.95	-0.484
AP1S2	7.97	1.53	4.22	1.00	2.65	2.45	4.93	2.62	5.35	6.41	6.19	7.95	6.30	12.27	5.65	-0.421
ARHGDIB	5.67	43.96	64.74	1.00	11.36	4.80	9.47	10.09	32.74	27.74	13.84	7.53	1.00	2.61	1.00	0.482
AURKB	6.89	1.65	11.97	1.86	8.65	8.72	2.78	2.38	17.63	11.87	2.03	2.95	4.60	1.48	1.00	0.570 *
ZFAND6	4.01	2.30	2.72	1.00	3.24	2.03	2.08	3.76	2.81	3.76	5.03	6.58	6.85	5.24	8.91	-0.545 *
BMI1	4.31	1.35	2.02	1.00	3.19	2.10	2.63	3.77	3.60	4.44	2.68	3.51	5.14	3.37	4.00	-0.316
BMP4	20.77	1.00	1.41	1.50	6.89	5.38	4.44	7.84	7.69	11.09	20.05	38.64	11.01	54.30	10.11	-0.457
CCNB1	4.68	1.00	6.51	1.03	4.05	5.07	2.18	2.10	8.81	9.14	2.47	3.56	3.50	1.35	2.30	0.441
CCND1	11.16	3.35	1.77	1.33	1.65	1.00	4.68	4.10	5.83	3.24	9.77	11.15	25.58	10.42	9.60	-0.573 *
CD74	49.05	108.12	475.17	60.81	127.06	98.06	266.55	208.06	9.45	130.24	5.76	13.36	2.77	1.00	19.62	0.565 *
CD97	6.79	2.28	3.64	1.00	2.47	1.94	2.82	2.51	5.89	4.10	1.55	5.57	7.79	4.26	1.14	-0.142
CDC20	7.80	4.37	11.94	1.00	7.19	9.13	3.35	2.71	15.51	12.58	4.37	5.16	5.62	1.19	2.30	0.466
CDKN2D	107.42	1.00	72.35	14.56	79.48	70.70	33.28	51.29	99.08	98.45	1.83	1.66	3.45	3.96	3.30	0.577 *
CDKN3	8.28	3.07	6.28	1.00	3.76	4.85	2.07	1.44	9.89	8.13	15.78	18.46	21.38	6.72	12.06	-0.437
COL7A1	11.63	2.58	4.81	1.72	3.79	3.60	3.03	7.97	8.47	10.19	1.53	4.08	3.69	1.00	2.76	0.216
CTGF	2.55	7.50	2.29	2.29	3.30	3.97	4.23	4.70	4.17	3.91	1.50	1.84	1.00	4.31	2.55	-0.094
DNCI1	12.67	10.94	17.47	3.39	15.32	7.22	19.86	15.11	23.86	20.00	3.53	2.65	1.00	3.44	5.53	0.451
DPYSL3	3.31	2.61	4.28	1.00	4.89	2.44	4.43	5.37	3.98	5.48	3.27	2.88	2.34	1.29	4.18	0.245
E2F1	5.83	1.74	7.37	1.00	4.16	4.82	2.75	2.56	7.44	6.49	1.52	1.76	2.64	1.94	1.41	0.566 *
EDG2	20.15	1.93	3.70	1.00	3.47	3.22	4.26	4.06	4.56	7.63	15.68	17.90	17.68	12.51	8.21	-0.497
EDG7	371.40	1.00	1.00	3.44	4.57	1.00	3.32	4.51	1.00	13.67	80.83	87.69	33.90	1.00	6.04	-0.123
EFEMP1	3.15	5.06	24.85	5.28	8.41	10.34	3.99	12.75	6.85	2.85	1.25	3.69	1.00	2.95	3.62	0.637 *
EIF4G2	4.47	3.51	3.33	1.00	3.13	2.56	2.64	3.88	3.82	4.45	6.75	7.95	9.10	6.89	9.01	-0.623 *
F2R	40.85	13.19	1.00	1.58	2.03	5.28	3.42	3.98	2.05	3.30	88.86	192.07	204.62	462.44	196.32	-0.530 *
MCTP2	33.06	1.00	1.78	1.71	21.05	9.17	10.07	12.25	12.51	8.15	40.17	119.57	63.25	10.97	38.79	-0.445
FST	10.93	1.29	1.69	1.00	1.77	1.65	2.49	4.09	3.26	5.36	10.10	19.58	22.05	37.80	11.17	-0.531 *
GABRB1	1.00	1.00	1.00	1.00	1.00	1.00	1.00	1.00	1.00	1.00	3.26	6.33	1.00	1.01	2.57	-0.380
GMFG	51.36	12.53	282.29	1.00	13.14	23.92	23.65	7.36	10.11	17.15	11.10	53.57	23.82	32.27	50.34	0.479
GPR37	22.59	1.00	25.67	11.43	25.68	21.54	22.46	33.71	55.00	51.26	22.22	3.02	4.02	1.00	1.00	0.436
HGF	5.25	2.65	4.05	1.00	6.46	3.01	7.68	3.22	4.60	7.11	3.88	6.21	2.36	6.12	2.79	0.023
HLA-DRA	420.47	813.77	7043.76	891.34	3723.59	1555.50	8657.26	4388.44	41.46	1301.63	37.92	96.69	1.00	12.90	192.60	0.391
HLA-DR3	1.00	33.48	1.83	56.93	180.53	9.18	763.47	45.31	1.42	32.79	1.00	1.00	1.00	1.00	1.00	-0.039
HNRPU	5.27	2.32	4.50	1.00	3.27	3.02	2.88	3.93	4.31	4.87	5.29	4.86	5.05	6.04	5.12	-0.357
ICAM1	14.19	43.36	36.65	3.89	97.90	14.87	35.24	62.73	12.91	35.19	5.44	26.02	1.00	38.10	14.83	0.343
IFI44	3.67	1.69	4.73	1.00	4.39	3.77	2.96	2.18	3.81	9.32	4.49	4.91	4.07	4.38	2.73	0.172
IGF1	1.00	1.32	1.57	2.37	8.79	4.09	4.96	8.18	2.35	6.59	5.59	6.41	1.28	8.49	6.32	-0.136
IGFBP5	6.44	3.78	6.67	1.03	1.62	1.00	4.75	2.44	4.15	6.28	43.24	47.49	16.77	50.54	89.51	-0.523 *
IGFBP7	1.68	1.46	2.79	0.85	3.43	3.38	1.00	1.97	1.85	4.32	1.83	2.47	1.58	6.09	2.27	0.072
IL13RA2	55.24	1.00	13.19	2.43	3.25	7.20	78.01	28.86	61.36	46.75	11.95	13.00	9.08	2.52	13.50	-0.049
INPP5E	8.15	1.94	7.55	1.84	5.27	3.58	3.96	6.94	7.28	8.61	1.65	4.48	4.57	3.70	1.00	0.428
ITGA3	19.98	8.16	13.13	1.00	5.96	4.35	14.70	7.32	12.35	19.41	4.82	6.29	8.10	6.44	18.15	0.017
ITGA5	3.70	5.12	5.39	1.35	6.20	3.98	5.70	7.94	5.85	6.80	1.00	1.64	1.46	1.00	1.89	0.402
ITGA6	71.48	5.15	4.44	1.00	10.67	10.58	36.96	51.64	121.51	103.91	106.94	94.41	77.33	18.30	114.62	-0.412
KCNK12	1.00	1.00	1.00	1.00	1.00	1.00	1.00	1.00	1.00	1.00	2.21	13.13	1.00	1.00	4.80	-0.327
KCNN2	6.80	1.00	1.00	1.00	1.00	1.00	1.00	1.00	1.19	1.21	16.88	6.09	6.14	1.00	1.00	-0.361
KCTD12	6.01	3.03	3.44	1.00	4.49	1.61	4.24	2.77	3.17	6.17	3.87	2.91	4.76	1.81	2.78	0.082
LAMA3	9.00	1.00	1.26	3.12	4.34	2.21	4.40	7.85	3.66	7.45	3.92	2.75	7.80	2.36	4.91	-0.143
LEPR	18.90	1.21	21.25	1.00	1.96	3.17	2.46	2.00	1.38	2.07	36.03	44.00	21.74	122.06	23.23	-0.369
LIF	61.87	19.90	60.39	10.39	65.84	43.08	38.59	32.33	66.66	74.06	8.82	5.70	21.43	27.65	1.00	0.603 *
LRP8	11.43	2.65	2.64	1.32	6.00	2.68	5.83	6.80	10.83	8.00	1.07	2.70	1.89	1.00	2.00	0.204
MCAM	30.70	41.87	197.66	24.55	63.88	56.08	222.30	151.21	183.32	212.27	1.64	7.90	1.00	2.68	30.82	0.414
MCM5	5.43	3.83	6.46	1.00	3.96	4.92	2.45	2.17	8.18	7.48	3.68	5.08	5.90	4.49	3.47	0.158
MCM7	7.26	4.18	6.76	1.00	4.07	5.64	2.89	2.99	8.01	7.55	2.07	3.34	3.50	2.22	2.75	0.375
TAF1D	5.71	1.25	2.68	1.00	2.96	2.69	3.13	3.47	3.99	4.11	1.71	2.97	2.22	2.12	2.34	0.100
MGP	144.38	117.61	152.97	82.08	933.56	49.38	196.27	631.65	35.40	202.33	289.56	331.07	1.00	109.25	235.89	0.241
MICA	2.71	1.20	2.61	1.00	2.82	1.70	1.73	3.82	6.11	2.67	1.91	1.31	1.46	4.28	3.31	0.047
NDUFA8	4.19	2.92	4.38	1.00	3.62	2.04	3.79	3.52	5.21	5.22	8.89	11.86	11.62	12.14	13.29	-0.577 *
NFE2L3	8.92	9.18	20.47	1.00	6.59	5.51	6.81	3.37	9.88	6.13	6.89	16.89	11.04	6.15	1.95	0.191
NPR3	14.70	7.58	50.15	7.67	6.65	16.67	10.03	7.78	10.74	8.41	3.26	4.09	2.16	31.52	1.00	0.464
NT5E	15.55	3.26	4.26	1.00	3.19	2.48	4.50	3.16	9.67	9.34	11.32	16.82	16.41	9.95	10.01	-0.486
P4HA2	2.36	3.46	2.59	3.74	13.95	4.93	13.14	16.28	15.83	13.93	1.14	1.04	1.59	1.00	5.69	0.253
PGR	73.41	1.00	2.24	1.39	4.12	1.00	4.31	1.31	4.51	1.73	8.93	9.70	7.58	1.00	1.45	-0.054
PGRMC2	6.58	3.88	3.63	1.00	3.51	2.87	3.60	5.31	3.31	3.35	8.24	13.82	9.27	16.27	12.28	-0.629 *
PLAU	4.40	11.43	4.77	1.00	6.96	3.47	2.42	4.50	1.70	1.68	12.09	13.10	9.42	4.59	10.96	-0.501
PLEC1	5.53	4.01	3.64	1.00	1.83	1.82	2.96	3.83	4.19	4.81	2.63	3.35	4.19	2.83	4.31	-0.249
PQBP1	5.95	3.18	4.98	1.00	3.71	3.47	3.22	4.29	5.01	6.85	9.32	13.82	11.20	14.40	13.32	-0.566 *
PRG1	2.39	5.57	5.93	1.00	3.50	2.37	5.39	5.11	4.19	3.87	1.06	1.47	2.14	2.93	12.91	-0.073
PRKAG1	5.85	1.44	5.59	1.00	4.46	2.75	3.96	4.56	5.38	6.84	3.32	4.97	4.17	3.21	4.01	0.204
PROS1	6.17	1.00	4.17	2.43	9.80	5.39	3.66	9.79	3.75	6.81	3.20	5.36	1.26	9.93	6.53	0.097
PSMC5	5.76	1.00	6.34	1.19	5.14	3.66	4.44	5.32	7.44	8.42	1.92	3.15	2.35	2.60	2.27	0.470
RAB3B	40.65	1.00	1.94	1.23	3.17	3.43	5.77	4.94	9.24	10.33	6.56	11.33	27.96	7.49	5.40	-0.237
RRM2	6.03	1.35	8.26	1.00	5.56	6.02	2.09	2.06	9.14	7.52	3.34	3.68	5.71	1.65	2.21	0.474
SERPINI1	10.07	1.00	1.06	1.79	4.60	2.56	8.73	7.27	8.94	10.47	16.09	20.40	7.85	23.14	23.11	-0.601 *
SLC16A4	8.14	3.04	6.95	1.00	2.87	2.17	4.42	3.30	3.28	5.60	4.93	7.83	7.41	2.26	3.91	-0.097
SLC20A1	7.63	6.95	3.57	1.00	4.29	3.26	6.38	4.17	8.01	6.68	7.09	8.66	10.81	3.91	10.23	-0.575 *
SLC2A1	4.14	6.97	6.91	1.15	6.43	4.26	3.88	5.47	6.10	3.75	1.02	1.25	2.15	1.06	1.00	0.492
SLCO1A2	1.00	1.00	1.00	1.00	1.00	1.00	1.00	9.29	1.00	1.00	1.00	1.00	1.00	86.08	1.00	-0.247
SPRY2	7.02	2.00	4.36	1.00	2.39	1.88	2.31	2.45	3.96	5.14	4.68	4.89	6.78	6.62	2.75	-0.243
TCF19	3.68	2.97	4.09	1.00	3.21	3.58	1.98	1.79	7.14	6.16	2.62	3.63	6.73	2.77	3.01	0.054
TFPI2	3.15	8.97	9.71	1.50	2.74	1.00	8.34	2.24	8.92	8.79	3.31	6.23	1.39	25.75	4.08	-0.123
TGM2	52.63	209.29	291.55	20.15	164.81	87.19	53.38	77.69	60.23	50.16	5.42	3.21	1.00	7.40	36.04	0.603 *
TIMP3	2.78	1.95	1.00	2.34	3.14	2.96	3.02	5.35	4.05	8.20	5.06	7.20	17.35	35.02	7.46	-0.407
TK1	10.24	3.60	10.30	1.00	5.25	5.42	3.30	2.12	11.22	8.43	3.34	7.28	7.63	2.48	2.72	0.318
TRIB2	4.41	2.96	4.73	1.00	4.75	3.48	2.99	2.96	4.11	5.57	3.35	3.07	1.79	6.71	2.99	0.178
TRIP13	5.92	1.00	6.74	1.11	4.13	5.18	2.15	2.36	8.28	7.58	2.70	3.24	4.93	1.58	2.06	0.425
UBE2C	4.94	1.11	7.87	1.00	5.72	7.90	2.40	2.11	10.38	9.49	2.88	3.51	3.34	1.03	1.54	0.500
UBE2S	8.37	3.31	5.83	1.00	7.43	5.82	3.95	5.27	10.16	11.40	4.16	6.44	6.95	3.41	4.42	0.203
UCHL1	12.47	4.12	4.76	1.00	4.65	3.78	5.89	6.74	8.12	8.59	7.90	13.48	10.88	7.50	17.84	-0.510
VLDLR	6.90	5.65	4.56	1.00	6.91	3.29	3.50	22.63	6.26	3.86	4.63	11.99	15.56	6.11	12.40	-0.399
WIF1	2.93	1.00	1.92	1.00	1.59	1.20	1.00	1.68	1.14	5.09	73.73	154.46	1.00	3.14	1.00	-0.320
ZNF185	3.92	2.23	4.95	1.00	3.83	3.67	8.28	5.68	8.20	32.11	4.75	3.81	4.04	3.38	4.67	0.167

***r*, Pearson correlation coefficient; ******P*<0.05**
